# P030. Global postural rehabilitation and migraine: a pilot-study

**DOI:** 10.1186/1129-2377-16-S1-A116

**Published:** 2015-09-28

**Authors:** Camilla Refice, Rita Loriga, Maria Novella Pompa, Giulia Gambale, Riccardo Altavilla, Matteo Paolucci, Claudia Altamura, Philippe Souchard, Fabrizio Vernieri

**Affiliations:** Centro di Rieducazione Posturale Globale, Casa di Cura Pio XI, Rome, Italy; Centro Cefalee, Unità di Neurologia, Università Campus Bio-Medico, Rome, Italy; Centre de Rééducation Posturale Globale, Saint-Mont, France

## Introduction

Global postural rehabilitation (GPR) is a method of physical therapy, designed by Professor Souchard, for the treatment of osteo-neuro-muscular pathologies. The correction of oculo-motor, cranio-cervical and temporo-mandibular joint dysfunctions inside “postural globality” can lead to the elimination of muscle tension that is one of the most important triggers and, at the same time, complications of headaches. The present study aimed at evaluating whether this method could be useful in reducing the number, intensity and duration of attacks and also the use of painkillers in patients with migraine without aura.

## Methods

We recruited a sample of 16 female patients, aged between 25 and 65 years, affected by migraine without aura. Following a randomized criterion, 8 patients were included in the “Experimental Group" and the other 8 patients in the “Control Group”. Both groups were evaluated before starting treatment (T0), after 3 (T1) and 7 weeks (T2). Intensity and quality of pain were evaluated by BS-11, PPI and SF-MPQ, while disability was assessed by BRS-6 and HIT-6. Experimental Group patients received a “Postural and Morfological Assessment” plus a particular evaluation of Oculo-Motor System, upper cervical district (C0-C2) and temporo-mandibular joint. This group underwent both adequate pharmacological treatment and 10 GPR sessions. Control Group received only pharmacological treatment. Each patient filled out a migraine diary: particular attention was paid to the number of painkillers taken.

## Results

*Friedman test* for non parametric data showed an improvement of all rating scales values in the Experimental Group. In particular, at T1 there was a decrease of all the considered parameters (pain intensity and quality, attacks duration, frequency and disability) compared to T0 (p < 0.05). Improvement trend resulted also at T2, except for two subjects (p < 0.05). In the Control Group, after an initial partial improvement at T1 compared to T0, most of the values remained unchanged or worsened; few patients improved at T2 compared to T1.

## Conclusions

Pain intensity and quality, attacks duration, frequency and disability improved in patients undergoing GPR. Furthermore, 80% of patients in the Experimental group replaced the anti-migraine medication, i.e., triptans, with NSAIDS, while the other 20% reduced the number of painkillers. Our study shows the efficacy of GPR treatment in patients affected by migraine without aura.

Written informed consent to publication was obtained from the patient(s).

